# *Mecp2*-Null Mice Provide New Neuronal Targets for Rett Syndrome

**DOI:** 10.1371/journal.pone.0003669

**Published:** 2008-11-07

**Authors:** Rocio G. Urdinguio, Lidia Lopez-Serra, Pilar Lopez-Nieva, Miguel Alaminos, Ramon Diaz-Uriarte, Agustin F. Fernandez, Manel Esteller

**Affiliations:** 1 Cancer Epigenetics Laboratory, Spanish National Cancer Research Centre (CNIO), Madrid, Spain; 2 Statistical Computing Team, Spanish National Cancer Research Centre (CNIO), Madrid, Spain; 3 Cancer Epigenetics and Biology Program (PEBC), Catalan Institute of Oncology (ICO) and Institut d'Investigacio Biomedica de Bellvitge (IDIBELL), Barcelona, Catalonia, Spain; 4 Institucio Catalana de Recerca i Estudis Avançats (ICREA), Barcelona, Catalonia, Spain; Italian National Research Council, Italy

## Abstract

**Background:**

Rett syndrome (RTT) is a complex neurological disorder that is one of the most frequent causes of mental retardation in women. A great landmark in research in this field was the discovery of a relationship between the disease and the presence of mutations in the gene that codes for the methyl-CpG binding protein 2 (MeCP2). Currently, MeCP2 is thought to act as a transcriptional repressor that couples DNA methylation and transcriptional silencing. The present study aimed to identify new target genes regulated by Mecp2 in a mouse model of RTT.

**Methodology/Principal Findings:**

We have compared the gene expression profiles of wild type (WT) and *Mecp2*-null (KO) mice in three regions of the brain (cortex, midbrain, and cerebellum) by using cDNA microarrays. The results obtained were confirmed by quantitative real-time PCR. Subsequent chromatin immunoprecipitation assays revealed seven direct target genes of Mecp2 bound *in vivo* (*Fkbp5*, *Mobp*, *Plagl1*, *Ddc*, *Mllt2h*, *Eya2*, and *S100a9*), and three overexpressed genes due to an indirect effect of a lack of Mecp2 (*Irak1*, *Prodh* and *Dlk1*). The regions bound by Mecp2 were always methylated, suggesting the involvement of the methyl-CpG binding domain of the protein in the mechanism of interaction.

**Conclusions:**

We identified new genes that are overexpressed in *Mecp2*-KO mice and are excellent candidate genes for involvement in various features of the neurological disease. Our results demonstrate new targets of MeCP2 and provide us with a better understanding of the underlying mechanisms of RTT.

## Introduction

Rett syndrome (RTT, OMIM 312750) is a complex and severe neurological disease. This X-linked disorder is the second most frequent cause of mental retardation in women, affecting one out of every 10,000–15,000 live births. After apparently normal development for 6–18 months, RTT girls lose their acquired cognitive, social, and motor skills [1], and develop autistic behavior accompanied by stereotypic hand movements. Further deterioration leads to severe mental retardation and motor impairments, including ataxia, apraxia, and tremors. Seizures, hyperventilation, and apnea are also common [2]. Male RTT patients have also been described; they usually suffer a more severe progression.

The development of this syndrome has been related to mutations in the gene encoding methyl-CpG-binding protein 2 (MeCP2) [3]. The MECP2 gene is located on Xq28 and consists of four exons spanning a region more than 75 kb long [4], [5]. Two isoforms of MeCP2 have been described to date [6], [7].

DNA methylation of specific sites in CpG islands is an important epigenetic mechanism in the regulation of some genes. Proteins with an affinity for methyl-CpGs, such as MeCP2, provide a link between DNA methylation and chromatin remodeling [8]–[11]. Moreover, there is growing evidence of a multifunctional role of MeCP2 [12] that is in accordance with its four different functional domains: (1) a methyl-CpG-binding domain (MBD), which accounts for MeCP2-specific binding to methyl-CpG pairs [13]; (2) an arginine-glycine repeat RNA-binding domain [14]; (3) a transcriptional repression domain (TRD) that interacts with a corepressor complex involving mSin3A and histone deacetylases [15]; and (4) an RNA splicing factor binding region (WW group II binding domain) [16].

In the first studies of MeCP2, abundant binding sites of this protein were found in genomic chromatin, and MeCP2 seemed capable of long-range repression [17]. Later it was involved in large-scale reorganization of pericentromeric heterochromatin during differentiation [18]. Although some reports initially suggested that MeCP2 action strongly influenced gene expression levels, more detailed analyses showed only subtle differences in expression patterns between wild type and MeCP2-deficient samples [19]. Despite the identification of a diverse set of target genes for MeCP2 [8], [20], [21], it remains unclear how MeCP2 dysfunction ultimately results in the neuronal syndrome. A very recent and surprising breakthrough in the field has been provided by Chahrour and co-workers [22], where the authors studied gene expression patterns in the hypothalamus of mice that either lack or overexpress MECP2 and observed that a majority of genes appeared to be activated by MeCP2. Thus, this study suggests that MeCP2 can function as an activator and a repressor of transcription [22].

Although Mecp2 is expressed in a wide range of tissues, the major features of RTT have already been reported as being mainly caused by the neuronal deficiency of Mecp2 [23], and for this reason we confined our study to the central nervous system (CNS). Given the difficulties of working with human samples, we chose a well-established mouse model of RTT that mimics the human disease [24]. The purpose of our study was to identify new target genes of Mecp2 in neuronal tissue from a mouse model of RTT, starting from an expression microarray approach and proceding with the validation of the target genes by single expression, chromatin immunoprecipitation and DNA methylation analyses. Using this strategy, we have unmasked previously uncharacterized genes that are disregulated in CNS upon Mecp2 disruption.

## Results

### Expression profile

In order to identify genes specifically regulated by Mecp2, we first carried out an expression-profile experiment in three separate brain regions (cortex, midbrain, and cerebellum), in an attempt to determine whether there was a brain region that was more sensitive to the lack of Mecp2 or that showed different regulation patterns. Total RNA was isolated from brain sections obtained from *Mecp2*-null (KO) mice and their wild type (WT) littermates. We performed direct competitive hybridization between WT and KO tissues, comparing each brain region separately. Four biological replicates were made, providing a total of twelve microarrays.

First, an ANOVA (analysis of variance) showed no significant differences between brain regions (ANOVA, adjusted p<0.05), indicating that CNS gene expression was homogeneous (with respect to the divisions made), thereby justifying the combined analysis of all the microarrays. Despite the sensitivity of the experiments and the large number of replicates, the significance analysis of microarrays [25] did not reveal any genes that were differentially expressed with respect to the false discovery rate (FDR). Although we found no significant differences, the microarrays yielded a list of genes whose expression was very likely to be affected by the lack of Mecp2. Globally there were few genes whose expression changed by >1.5-fold. Only 29 probes were upregulated in KO mice (two genes with at least twice the level of expression) (Table 1), and 24 downregulated (two genes with less than half the level of expression) (Table 2). The fact that *Mecp2* was as the most downregulated gene in KO mice confirmed that the experiments were reliable (Table 2).

**Table 1:** Probes with higher level of expression in KO samples than in WT controls. (upregulated), as determined by microarray analysis.

	Name	UniGene	GeneBank	Description	n-fold change	
**▸**	***Fkbp5***	Mm.276405	BG087373	FK506 binding protein 5	2.76	**◂**
**▸**	***Mt1***	Mm.192991	BG077818	Metallothionein 1	2.25	**◂**
**▸**	***Bai1***	Mm.43133	BC037726	Brain-specific angiogenesis inhibitor 1	1.99	**◂**
**▸**	***Mt2***	Mm.147226	BG063925	Metallothionein 2	1.94	**◂**
**▸**	***Pcolce2***	Mm.46016	BQ561584	Procollagen C-endopeptidase enhancer 2	1.90	**◂**
	*Pnpla2*	Mm.29998	BG077619	Patatin-like phospholipase domain containing 2	1.73	
	*Rpl36*	Mm.379094	BG072993	Sulfatase modifying factor 1	1.72	
**▸**	***Irak1***	Mm.38241	BG076768	Interleukin-1 receptor-associated kinase 1	1.71	**◂**
**▸**	***Mobp***	Mm.40461	AK013799	Myelin-associated oligodendrocytic basic protein	1.62	**◂**
			BQ550269		1.58	
**▸**	***Prodh***	Mm.28456	BQ553283	Proline dehydrogenase	1.55	**◂**
**▸**	***Dlk1***	Mm.157069	BQ550065	Delta-like 1 homolog (Drosophila)	1.55	**◂**
	*Rps15*	Mm.643	BG088213	Ribosomal protein S15	1.55	
**▸**	***Plagl1***	Mm.287857	BG085853	Pleiomorphic adenoma gene-like 1	1.54	**◂**
**▸**	***Ddc***	Mm.12906	BQ554377	Dopa decarboxylase	1.52	**◂**
**▸**	***Gas5***	Mm.270065	BG085421	Growth arrest specific 5	1.52	**◂**
**▸**	***Mllt2h***	Mm.6949	BQ554269	AF4/FMR2 family, member 1	1.52	**◂**
			AW555131		1.52	
**▸**	***Txnip***	Mm.271877	BG086605	Thioredoxin interacting protein	1.52	**◂**
**▸**	***Eya2***	Mm.282719	BG069346	Eyes absent 2 homolog (Drosophila)	1.51	**◂**
**▸**	***Cpm***	Mm.339332	BQ560184	Carboxypeptidase M	1.51	**◂**
**▸**	***Nudt9***	Mm.241484	BQ561264	Nudix (nucleoside diphosphate linked moiety X)-type motif 9	1.51	**◂**
	1700084E18Rik	Mm.297949	BG070023	RIKEN cDNA 1700084E18 gene	1.50	
	1700023B02Rik	Mm.292140	BG065239	RIKEN cDNA 1700023B02 gene	1.50	
**▸**	***Pxmp2***	Mm.21853	BG086336	Peroxisomal membrane protein 2	1.50	**◂**
	2010315L10Rik	Mm.41890	BG063434	RIKEN cDNA 2010315L10 gene	1.50	
	*Rpl18a*	Mm.379251	BG072556	Ribosomal protein L18A	1.50	
**▸**	***Ucp2***	Mm.171378	BG087187	Uncoupling protein 2 (mitochondrial, proton carrier)	1.50	**◂**
**▸**	***S100a9***	Mm.2128	BG072801	S100 calcium binding protein A9 (calgranulin B)	1.50	**◂**

Only probes with >1.5-fold change are shown.

Genes selected for subsequent assays are in bold and indicated by arrows (**▸ ◂**).

**Table 2:** Probes with lower level of expression in KO samples than in WT controls (downregulated), as determined by microarray analysis.

	Name	UniGene	GeneBank	Description	n-fold change	
	*Sqle*	Mm.296169	BG077950	Squalene epoxidase	0.67	
	*Rock1*	Mm.6710	BG088815	Rho-associated coiled-coil forming kinase 1	0.67	
			BG067593		0.67	
	*Scd2*	Mm.193096	BG066641	Stearoyl-Coenzyme A desaturase 2	0.66	
	5031439A09Rik	Mm.369129	BQ559666	RIKEN cDNA 5031439A09 gene	0.66	
	*Eif4g2*	Mm.185453	BG070960	Eukaryotic translation initiation factor 4, gamma 2	0.66	
**▸**	***Calb1***	Mm.277665	BG071405	Calbindin-28K	0.66	**◂**
	*Taf5l*	Mm.291777	CK335137	TAF5-like RNA polymerase II, p300/CBP-associated factor	0.65	
	*Tcea1*	Mm.207263	BG081444	Transcription elongation factor A (SII) 1	0.65	
**▸**	***Calb1***	Mm.277665	BG072229	Calbindin-28K	0.64	**◂**
			AW539369		0.63	
			BG087011		0.63	
**▸**	***Fabp7***	Mm.3644	AK021271	Fatty acid binding protein 7, brain	0.63	**◂**
			BQ551801		0.59	
	*Rhoa*	Mm.757	BG081880	Ras homolog gene family, member A	0.59	
			BQ551690		0.58	
			BG076236		0.58	
**▸**	***Sc4mol***	Mm.30119	BG083949	Sterol-C4-methyl oxidase-like	0.58	**◂**
		Mm.336117	BG081054	X-linked lymphocyte-regulated 3A	0.58	
			BG085378		0.57	
**▸**	***Itm2a***	Mm.193	BG088255	Integral membrane protein 2A	0.53	**◂**
			AW558689		0.42	
**▸**	***Gprasp1***	Mm.271980	AW553322	G protein-coupled receptor associated sorting protein 1	0.25	**◂**
**▸**	***Mecp2***	Mm.131408	BQ553027	Methyl CpG binding protein 2	0.05	**◂**

Only probes with <0.67-fold change are shown.

Genes selected for subsequent assays are in bold and indicated by arrows (**▸ ◂**).

**Figure 1:**
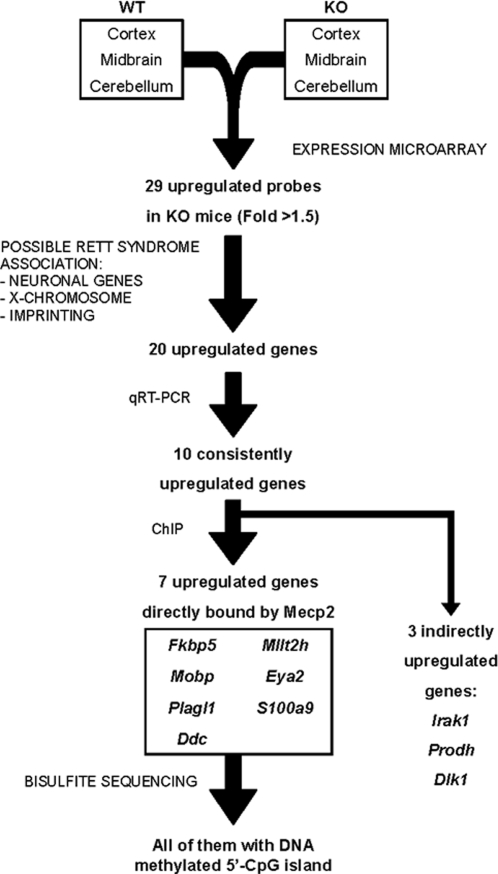
Schematic strategy used to identify new Mecp2 target genes in a mouse model of RTT.

Given that Mecp2 is known to be a transcriptional repressor and we were looking for direct targets of the protein, we focused our work on the genes that appeared to be upregulated in KO mice (Fig. 1). Moreover, we decided to study further those candidate genes from the microarray data that had been associated with neuronal function in published studies or that could be connected with Mecp2 function for their association with imprinting or X-chromosome inactivation. With that criterion we followed our study working with 20 genes, pointed by an arrow in Table 1. The second most down-regulated gene (*Gprasp1*) was chosen as a control gene for following assays (*Mecp2* was not appropriate as it was missing from KO mice) (Table 2).

### Validation of expression changes

**Figure 2:**
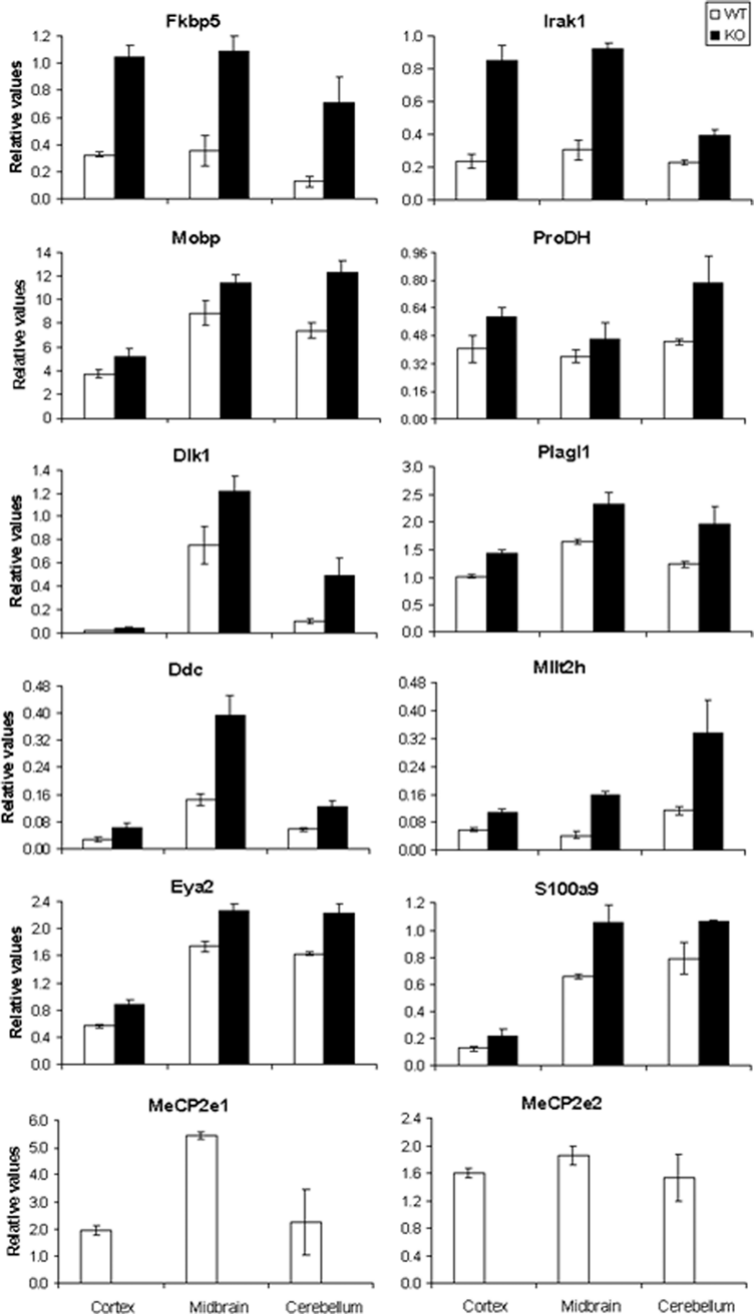
Relative expression results of each gene by qRT-PCR (normalized with respect to *Gapdh*). Genes confirmed to have significantly different expression in Mecp2-WT and KO samples are shown. White bars correspond to WT samples and black bars to KO. Values for each tissue are displayed separately. Data are from three independent biological replicates. Error bars indicate standard deviation (SD).

We first checked that the mouse model was working appropriately by testing the expression of the two Mecp2 isoforms described to date [7], [26]. Neither isoform could be detected in brain tissue from the KO mice, while the WT mice showed normal expression (Fig. 2 and Table 3), confirming that the model worked in accordance with our expectations.

**Table 3:** Significance (p-value) of Welch's t test comparing relative expression values of WT vs. KO tissues from each gene.

UPREGULATED GENES			
	Cortex	Midbrain	Cerebellum
***Fkbp5***	0.0001 ***	0.0158 *	0.0015 **
*Mt1*	0.0535	0.1844	0.0939
*Bai1*	0.0299 *	0.1142	0.0903
*Mt2*	0.0776	0.0191 *	0.0778
*Pcolce2*	0.1591	0.0064 **	0.0944
***Irak1***	0.0022 **	0.0087 **	0.0019 **
***Mobp***	0.0217 *	0.0378 *	0.0016 **
***ProDH***	0.0488 *	0.1732	0.0319 *
***Dlk1***	0.0017 **	0.0469 *	0.0029 **
***Plagl1***	0.0022 **	0.0148 *	0.0314 *
***Ddc***	0.0061 **	0.0011 **	0.0028 **
*Gas5*	0.8500	0.5158	0.0810
***Mllt2h***	0.0063 **	0.0047 **	0.0104 *
*Txnip*	0.0610	0.0380 *	0.2077
***Eya2***	0.0019 **	0.0014 **	0.0061 **
*CPM*	0.0693	0.1563	0.0502
*Nudt9*	0.2216	0.7973	0.8545
*Pxmp2*	0.3373	0.0474 *	0.1439
*Ucp2*	0.0632	0.6477	0.1687
***S100a9***	0.0256 *	0.0198 *	0.0728
*Gtl2*	0.5726	0.3441	0.3158

DOWNREGULATED GENES			
	Cortex	Midbrain	Cerebellum
***MeCP2e1***	0.0027 **	0.0010 ***	0.0210 *
***MeCP2e2***	0.0023 **	0.0009 ***	0.0119 *
***Gprasp1***	0.0323 *	0.0080 **	0.0321 *
***Itm2a***	0.0185 *	0.0050 **	0.0017 **
***Sc4mol***	0.0078 **	0.0459 *	0.1178
***Fabp7***	0.0205 *	0.0743	0.0088 **
***Calb1***	0.0086 **	0.3338	0.0405 *
***Sst***	0.0147 *	0.0148 *	0.0332 *
***Oprk1***	0.0981	0.0072 **	**0.0612**
*Gamt*	0.0419 *	0.2831	0.2741
***Creb1***	0.0016 **	0.0148 *	0.0258 *

Asterisks show the level of significance: * for p<0.05, ** for p<0.01, and *** for p<0.001.

Genes that appear in bold were consistently and significantly upregulated in at least two tissues and were selected for subsequent assays.

Among the down-regulated genes appear those ones selected from the article: Chahrour M, Jung SY, Shaw C, Zhou X, Wong ST, Qin J, Zoghbi HY (2008) MeCP2, a key contributor to neurological disease, activates and represses transcription. Science 320:1224–1229.

To validate the microarray results, we performed quantitative real-time PCR (qRT-PCR) of all the previously selected genes. We reverse-transcribed total RNA from all brain regions of three KO mice and their corresponding WT littermates. After normalizing the data with respect to their own *Gapdh* results, the expression values of all genes in the WT and KO mice were compared among the three brain regions separately. Statistical analysis (Welch's t test) of these data provided the p-values shown in Table 3.

Ten of the 20 upregulated genes tested were confirmed to be consistently upregulated in at least two of the three brain tissues by qRT-PCR. Eight genes (*Fkbp5*, *Irak1*, *Mobp*, *Dlk1*, *Plagl1*, *Ddc*, *Mllt2h*, and *Eya2*) had significantly stronger expression in KO mice in all the tissues studied. Additionally, two other genes (*Prodh* and *S100a9*) had a significantly higher level in two of the brain areas (cortex/cerebellum and cortex/midbrain, respectively) (Table 3). Given that severe symptoms of the RTT syndrom (anxiety, autonomic abnormalities, sleep-wake rhythm…) could be attributed to hypothalamic dysfunction and that this brain region undergo important gene expression changes upon Mecp2 impairment [22], we assessed the expression of our observed Mecp2-null mice upregulated genes in the hypothalamus. We found that these genes (*Fkbp5*, *Ddc*, *Dlk1*, *Irak1*, *Mllt2h*, *Mobp*, *Plagl1*, *Eya2*, *ProDH and S100a9*) where also all of them identified as upregulated in the hypothalamus of our MeCP2-null mice (Figure S1). To add internal methodological consistency to these data, we confirmed that those genes previously found downregulated in the hypothalamus upon Mecp2 disruption (such as Sst, Gamt, Creb1 and Oprk1) [22] were also found downregulated in the hypothalamus of our Mecp2 null mice (Figure S2). We also observed that Sst, Gamt, Creb1 and Oprk1 were downregulated in the cortex, midbrain and cerebellum of our Mecp2 null mice (Figure S2). The five most downregulated genes of our microarray study (*Gprasp1*, *Sc4mol*, *Calb1*, *Fabp7 and Itm2a*) were also significantly more weakly expressed in all the brain tissues studied in the Mecp2 null mice (Table 3 and Figure S3). *Fkbp5* has already been identified as an upregulated gene in the mouse model of Rett Syndrome [27] so can be considered as a positive control of the accuracy and appropriateness of the experiments.

It is remarkable that the expression of some genes (*Fkbp5*, *Irak1*, *Dlk1*, *Ddc*, and *Mllt2h*) in KO mice is twice that of the levels in WT mice, while the level of expression of other genes (*Mobp*, *Prodh*, *Plagl1*, *Eya2*, and *S100a9*) was 40–50% higher than those of WT values. Conversely, one of the overexpressed genes, *Dlk1*, is transcriptionally regulated by the methylation status of the CpGs upstream of *Gtl2* gene [28]–[30]. For this reason we tested *Gtl2* expression and confirmed that it did not change in KO mice with respect to WT levels (data not shown). Subsequent studies were made only with genes whose differences in levels of expression were confirmed by these assays (Fig. 1).

### Mecp2 direct binding to gene promoters

**Figure 3:**
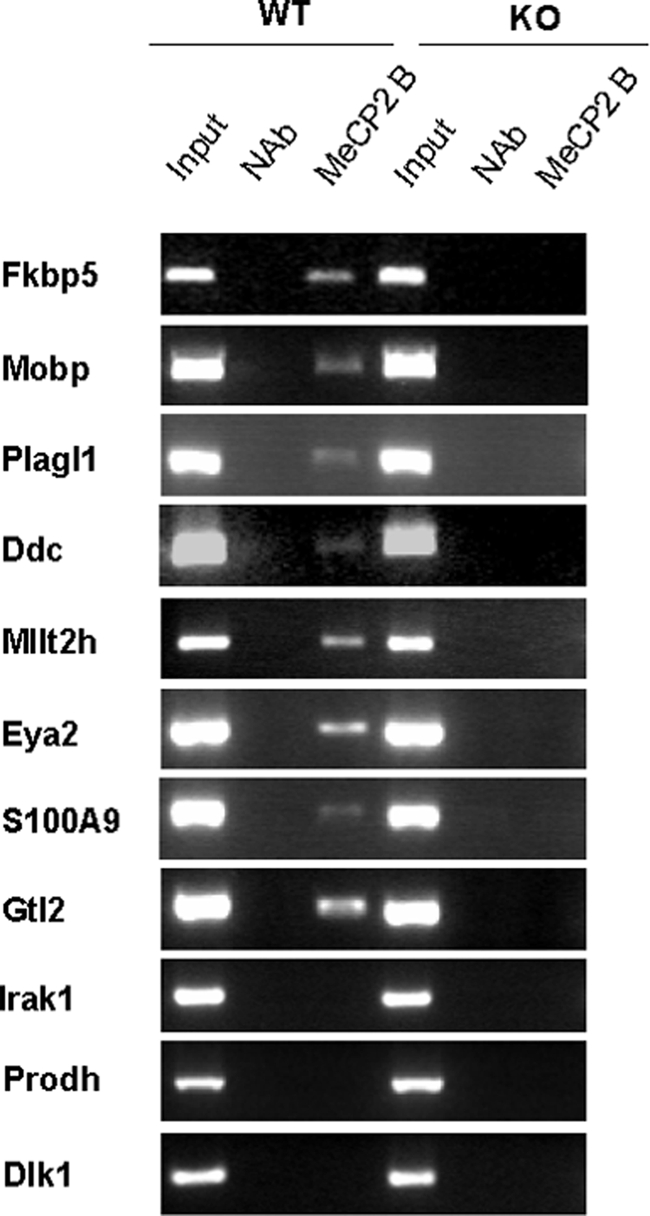
Results from chromatin immunoprecipitation assays. For *Mecp2*-WT and KO animals a fraction of total DNA (Input), a no-antibody control (NAB), and a fraction immunoprecipitated by the antibody (Mecp2 B) were tested. Three replicates of each reaction were performed.

Having completed the expression experiments, we wondered which genes were directly regulated by Mecp2. To reveal whether Mecp2 interacts with the gene promoters, we carried out conventional chromatin immunoprecipitation (ChIP) and quantitative-Chip (qChIP) assays. We tested the promoter regions of the genes confirmed by qRT-PCR and found that seven of the ten upregulated genes were bound to their promoter region by Mecp2 (*Fkbp5*, *Mobp*, *Plagl1*, *Ddc*, *Mllt2h*, *Eya2*, and *S100a9*) (Fig. 3 and Figure S4). However, although the region close to the *Dlk1* transcriptional start site was not bound by Mecp2, this gene has been described as being regulated by the region upstream *Glt2* [28]–[30]. Therefore, to confirm possible regulation by Mecp2, we checked the region upstream of *Gtl2* and found that it was bound by Mecp2 (Fig. 3 and Figure S4). For the downregulated genes *Gprasp1*, *Sc4mol*, *Calb1*, *Fabp7 and Itm2a* (Fig. 3 and Figure S3), we did not observe Mecp2 occupancy in their corresponding 5′-regulatory regions (Figure S3).

Conversely, we also found two genes, *Irak1* and *Prodh*, whose expression was indirectly affected by the lack of Mecp2, because this protein did not interact with their promoter regions (Fig. 3 and Figure S4).

### DNA methylation analysis of particular genes

**Figure 4:**
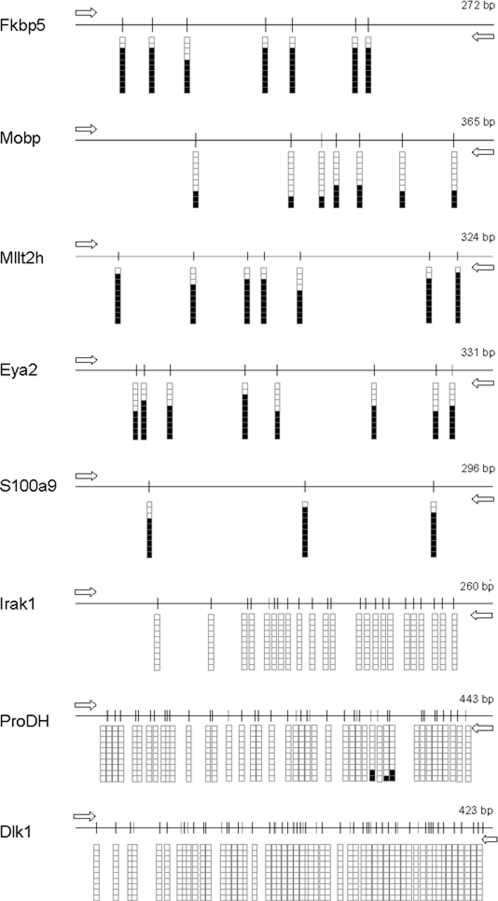
Plots representing bisulfite genomic sequencing results for the 5′-regions of the upregulated genes identified in Mecp2 null mice. Each shows a cloned fragment and the CpGs included. Ten clones are shown for every gene in which one column represents a CpG. Given that no differences were found between tissues or samples, one representative sample is shown for each gene. White squares correspond to non-methylated CpGs and black squares to methylated CpGs.

As Mecp2 is a methyl-CpG binding protein, we decided to explore the methylation status of gene promoters overlapping the Mecp2-bound regions. We extracted DNA from the three different brain regions of WT and KO mice and performed bisulfite sequencing (BS) analyzing ten clones for each condition. Statistical analyses of the resulting percentages of methylation showed no strong evidence of differences between the brain regions studied (ANOVA –see Methods–, p = 0.076). Moreover, there were no modifications in the DNA methylation pattern between WT and KO mice (ANOVA –see Methods–, p = 0.688), as Mecp2 has not been proposed to alter DNA methylation. BS results from one representative sample of each gene are shown in Fig. 4. From the ten confirmed upregulated genes, seven were methylated promoters, corresponding to *Fkbp5*, *Mobp*, *Plagl1*, *Ddc*, *Mllt2h*, *Eya2*, and *S100a9*. While *Dlk1* promoter was unmethylated, the CpGs upstream *Gtl2* (corresponding to the Mecp2-bound region) was methylated, which implies that this region is responsible for Mecp2 regulation of *Dlk1* expression. For the imprinted genes *Plagl*, *Ddc* and *Gtl2*, an extended analysis of twenty clones from bisulfite genomic sequencing rendered approximately a 50% unmethylated / 50% methylated sequences, as expected (Figure S5). In the end, all the gene promoters that were directly bound by Mecp2 were methylated, whilst the 5′-ends of Irak1 and ProDH (where Mecp2 was not bound) were unmethylated (Figure 4). In comparison, the five downregulated genes identified upon Mecp2 disruption (*Gprasp1*, *Sc4mol*, *Calb1*, *Fabp7 and Itm2a*) were all of them unmethylated, in concordance with the results recently described by Chahrourt et al. [22]

## Discussion

The purpose of this study was to identify new direct target genes of Mecp2 that could explain the development of RTT. To address this matter we first compared the RNA expression patterns of KO mice and their WT littermates using an expression microarray approach. We analyzed three brain regions but the microarray results did not show gene expression differences between the tissues. Nevertheless the expression profile comparisons highlighted a group of genes with altered expression in KO mice that exceeded a 1.5-fold change threshold. Consistent with previous reports [19], [20], [31] only a few genes showed a difference in expression in the absence of Mecp2, which is one of the most striking features of RTT.

We concentrated our study on genes that were upregulated in KO mice, given that our purpose was to find direct target genes of the transcriptional repressor Mecp2. Focusing our study on genes that could be relevant to RTT, qRT-PCR validated ten genes as being consistently upregulated in KO mouse brains. Subsequent ChIP assays showed that three of these genes (*Irak1*, *Dlk1* and *Prodh*) were upregulated, probably due to an indirect effect of the lack of Mecp2. On the other hand, we found that seven of the consistently upregulated genes (*Fkbp5*, *Mobp*, *Plagl1*, *Ddc*, *Mllt2h*, *Eya2*, and *S100a9*) were directly bound by Mecp2 to their promoter or regulatory region (Fig. 1).

The use of BS to characterize the DNA regions bound by Mecp2 revealed no methylation differences between brain regions or between KO and WT samples, so the expression differences were a direct consequence of the absence of Mecp2. Moreover, the regions bound by Mecp2 were always methylated, suggesting that the regulation carried out by Mecp2 might involve the MBD domain of the protein. It is not well known how the absence of Mecp2 would give rise to an increased expression at the DNA-methylated genes, but a loss of Mecp2-mediated recruitment of other partners of the chromatin and epigenetic machinery (such as histone deacetylases and histone methyltransferases) might be involved. In this regard, depletion of Mecp2 in human cancer cells by RNA interference is also able to partially induce release of gene silencing of DNA methylated CpG islands in association with a shift in the histone modification pattern towards a more permissive state of transcription [32].

Several expression profiles involving *Mecp2*-null samples have been performed to date. Despite the different tissues and specimens used, only small differences in expression patterns were found. Two different approaches were adopted in studies of human lymphocytes from RTT patients. First, the work of Delgado *et al.* [33] avoided mosaicism by using lymphoid clones, while, second, the study of Ballestar *et al.* [34] worked with non-subcloned samples to approximate physiological conditions more closely. In both cases, the decision to analyze lymphocytes was useful, given that they are accessible and are not invasive for patients. Another study used fibroblast strains from patients [35]. However, as RTT features seem to be caused mainly by a Mecp2 defect in the brain [23], it is possible that the peripheral deregulation may not be equivalent to that happening in the CNS and, unsurprisingly, these studies do not identify the same deregulated genes as in the present work.

Nevertheless, the work performed with post-mortem RTT brains [36] found a higher level of S100A9 expression, which is consist with our results (Table 1). These data support our findings, and it is possible that this study did not find more genes in common because of the mosaicism of human samples, which could have masked the subtle expression differences that we have detected.

The study of Tudor *et al.* [19] was carried out with brain samples in a different mouse model of RTT [23] and no genes were found in common with ours, possibly due to their different approach. Their work examined changes associated with disease progression before and after the onset of RTT symptoms. As they did not find any significant differences in expression they looked for predictor genes that could classify their samples. Given that their purpose and approach differed from ours, it is not surprising that they obtained different results from us.

Finally, it is essential to compare our results with those from studies performed in the same mouse model of RTT as investigated here. We found *Fkbp5* among the genes that are regulated by Mecp2 through the techniques of the microarray, qRT-PCR, ChIP, and BS. Given that *Fkbp5* is already known to be an upregulated gene in this RTT mouse model [27], it represents a good control of the accuracy of all the experimental assays. Moreover, a recent publication by Jordan *et al.* [31] showed that *Irak1* was more strongly expressed in cerebellum, but here we show that this upregulation is common to all KO brain tissue. However, there is some controversy regarding *Gtl2*. On one hand, Kriaucionis *et al.* [37] detected higher *Gtl2* levels in whole brain samples from late symptomatic mice, but on the other, another report [31] could not confirm differences in *Gtl2* expression between WT and KO in cerebellum or forebrain using qRT-PCR. Our results clearly showed no difference in *Gtl2* levels between any brain regions in WT and KO mice. We found that Mecp2 binds upstream of *Gtl2* but we suggest that this affects *Dlk1* expression according to the regulation previously described for this region [28], [29].

The importance to RTT of the genes identified in our work becomes evident from a detailed study of each of them individually. First, three genes regulated by Mecp2 are imprinted, a process in which Mecp2 had been already implicated [38], [39]. One of those genes, *Dlk1*, is a paternally expressed gene that encodes a protein homologous to the Notch/Delta family. Dlk1 is active both as a soluble and a transmembrane protein, and plays roles in the differentiation of several tissues [40]–[43]. In fact, a recent study related its function with ventral midbrain-derived dopaminergic precursor differentiation [44]. An intergenic differentially methylated region located 13 kb upstream of *Gtl2* regulates expression of the imprinted domain of murine distal chromosome 12 [28], [29]. Therefore, we verified that Mecp2 binds the sequence responsible for *Dlk1* expression without changing the levels of *Gtl2*. Our study identified a new partner of the mechanism involved in the regulation of imprinting of this region that may also be involved in neuronal differentiation.

The other imprinted gene, *Plagl1* (*Zac1*/ *Lot1*), is located in human and mouse chromosomal regions that are maternally imprinted [45], [46], and seems to control cell fate during neurogenesis, chondrogenesis, and myogenesis [47]. Nevertheless, our results may indicate the existence of a more complex network, given that Plagl1 acts as a transcription factor [48]. It has been demonstrated that *Plagl1* re-expression in a neuroblastoma cell line induces several imprinted genes, including *Dlk1* [49]. Consequently the upregulation of *Dlk1* could be caused by a double pathway; one involving the missing regulation of Mecp2, and the other involving *Plagl1*.

Other Mecp2-target genes are related to a wide spectrum of functions. For instance, Mobp is one of a family of oligodendrocyte-specific polypeptides. Several Mobp features suggest a possible key role in CNS biogenesis [50]. Intriguingly, however, *Mobp*-null mice lack any clinical phenotype [51]. Further research into this protein is required in order to identify its function and mechanism of action, and thereby assess the relevance of its upregulation to RTT pathology.

L-dopa decarboxylase (Ddc) is involved in the production of monoamine neurotransmitters and it is the rate-limiting enzyme for the synthesis of trace amines in mammals [52], [53]. Although Ddc activity is modulated by several factors [54]–[56], little is known about its gene regulation. Moreover, this enzyme's involvement in the pathology of neoplastic, neurological, and psychiatric disorders [57]–[59] may be related to RTT symptoms.

*Mllt2h*, also known as *Af4*, is a member of a family of four proline- and serine-rich proteins whose conserved ALF domain is thought to act as a transcriptional factor [60]. Detailed research with the robotic mouse model has shown that the regulation of the Af4 family is important for the normal function of the CNS [61]. Moreover, robotic mutant Af4 is deficiently degraded [61] and, as a result, an increased amount of the protein interacts with its targets. Our *Mecp2*-null mice model showed stronger *Mllt2h* (*Af4*) expression; we propose that it could share characteristics with the robotic mouse based on the large amount of Af4 present. Obviously, further research is required to investigate this hypothesis.

Mammalian Eya (*Eyes Absent*) proteins are predominantly cytosolic proteins that interact with members of the Six family of transcription factors. This interaction facilitates the translocation of Eya into the nucleus, where it serves as a coactivator of Six in the regulation of downstream genes [63] controlling precursor cell proliferation and survival during mammalian organogenesis [64]. However, Eya2 also binds selectively to some forms of active heterotrimeric Gαi proteins, preventing its translocation into the nucleus and avoiding Eya2/Six4-mediated transcription [65]. All these phenomena require careful exploration in order to determine thoroughly their possible involvement in brain development and in RTT.

S100 proteins are the largest family within the EF-hand protein superfamily. They are small acidic proteins exclusive to vertebrates that display some unique features not present in other EF-hand proteins [66]. Moreover, the heterodimers of S100A8 and S100A9 are the main calcium-binding proteins in phagocytes, where they regulate migration-modulating tubulin polymerization [67]. Nevertheless, complex, extracellular regulatory activities of the S100A8/S100A9 heterodimer, and, possibly, different functions of individual S100A9 and S100A8 homodimers have been suggested [68]. In any case, calcium is a ubiquitous second messenger that regulates a wide range of cellular events. Different calcium signals would lead to cellular changes involving calcium-binding proteins such as the S100 family. The effects of the overexpression of S100a9 in RTT pathology require further analysis.

Finally, two genes were upregulated due to an indirect mechanism caused by the lack of Mecp2. The first one, *Irak1* is located downstream of *Mecp2* in mouse (X29.6cM) and human (Xq28). Its overexpression may be due to a negative regulator loss or a chromatin structure abnormality caused by the deletion produced by knocking out *Mecp2*. This is currently the most plausible explanation, given that other RTT mouse models have been analyzed but not have not shown *Irak1* deregulation. However, large deletions of *MECP2* including *IRAK1* have already been reported in RTT patients [69]. A more profound study of their relationship could lead to a better understanding of the chromatin regulation of this region and might explain some features of the disease. The second gene, *Prodh*, encodes proline oxidase (POX), a mitochondrial inner-membrane flavoenzyme that is expressed in brain, liver, and kidney. This enzyme catalyzes the rate-limiting step of proline degradation [70]. This reaction, apart from being related to glutamate and ornithine synthesis, can transfer redox potential between subcellular compartments and between cells [71], [72]. Furthermore, proline protects against oxidative stress, and overexpression of POX results in decreased cell survival [73]. Several studies of Prodh deficiency have associated mutations in its gene with cognitive defects, autistic behavior and epilepsy [74]–[76], which underlines its importance in proper brain function. Additionally, proline metabolic properties in the CNS suggest that this aminoacid can act as an inhibitory neurotransmitter and/or as a metabolic precursor of glutamate in subpopulations of glutamatergic neurons [71], [72], [77]. For these reasons we hypothesize that *Prodh* overexpression and its corresponding increased catalytic activity could lead to a condition of neurotransmitter imbalance in the brain that might be accompanied by reduced cell survival.

In summary, all the genes reported in our study are of use to us for gaining a better understanding of the development and progression of RTT. The diversity of the pathways involved attest to the great complexity of this disease. Further studies are necessary to extend our knowledge of the deregulated network of genes and affected mechanisms that are involved in RTT.

## Materials and Methods

### Animal model

B6.129P2(C)-*Mecp2^tm1.1Bird^*/J (stock number: 003890) heterozygous females (*Mecp2* ^+/−^) and wild-type females were obtained from the Jackson Laboratory (Bar Harbor, ME). Briefly, the mutant strain was generated by replacement of exons 3 and 4 of *Mecp2* in embryonic stem cells with the same exons flanked by loxP sites. The deletion of the gene was achieved by crossing *Mecp2* ^lox/lox^ females with mice with ubiquitous Cre expression [24]. *Mecp2* ^+/−^ females were mated with C57BL/6J males and their offspring were genotyped by PCR according to the supplier's protocol (http://jaxmice.jax.org/strain/003890_3.html).

### Mouse housing and sample collection

Animals were kept under specific pathogen-free conditions in accordance with the recommendations of the Federation of European Laboratory Animal Science Associations. Mice were inspected daily and maintained under controlled lighting conditions (lights on from 08:00h to 18:00h), at constant temperature (22°C), and were allowed *ad libitum* access to food and water.

All experiments were performed in hemizygous *Mecp2*-null males (*Mecp2* ^−/y^) aged 6–10 weeks. Mice were euthanized in accordance with the Guidelines for Humane Endpoints for Animals Used in Biomedical Research. Samples were obtained from KO mice and their WT littermates after the establishment of RTT symptoms in the defective animals. Brains were removed and divided into cortex, midbrain, and cerebellum. The brain regions were dissected, immediately frozen on dry ice, and stored at −80°C until use, except for tissue used ChIP assay samples, which was freshly processed. WT and KO mouse tissues were extracted and handled under identical conditions.

### RNA isolation, amplification, and hybridization

Tissue samples were homogenized in Trizol reagent (Invitrogen Corp., Carlsbad, CA). Phase separation was done following the manufacturer's instructions (http://www.invitrogen.com/content/sfs/manuals/15596026.pdf). RNA was preferentially purified using the RNeasy Mini kit (Qiagen Inc., Valencia, CA). RNA was cleaned up by RNase-free DNase I treatment (Qiagen Inc.). Total RNA was quantified by spectrophotometry and quality was verified on an agarose gel.

For amplification, 4 µg of total RNA were first reverse-transcribed using T7-oligo (dT) promoter primer, followed by RNase-H-mediated second-stranded cDNA synthesis (Invitrogen Corp.). Double-stranded cDNA was purified and served as a template in the subsequent RNA polymerase amplification (MEGAscript T7, Ambion Inc., Austin, TX). Amplified RNA was purified using RNeasy Mini kit (Qiagen Inc.), then quantified and its quality confirmed as for the total RNA.

Amplified RNA was reverse-transcribed and labeled using random hexamers (Promega) and Cy3 or Cy5-dUTP (Amersham, Piscataway, NY), depending on the sample (control or *Mecp2*-null tissue, respectively). After degradation of template RNA, every labeled sample was purified using the Cyscribe GFX Purification kit (Amersham). Samples for comparison were mixed in pairs in order to carry out a direct competition hybridization experiment, then combined with SlideHyb Buffer (Ambion), mouse Cot-1 DNA (Invitrogen Corp.), RNA polyA (Sigma-Aldrich, St Louis, MO), and yeast tRNA (Invitrogen Corp.). Samples were subsequently hybridized to CNIO mouse cDNA microarrays.

### Microarray analysis

Briefly, the CNIO mouse cDNA microarray contains both the NIA 15 K and 7.4 K clone sets from the National Institute on Aging (http://lgsun.grc.nia.nih.gov/cDNA/cDNA.html), and an additional 600 clones specifically associated with cancer, angiogenesis, apoptosis, signal transduction, and stress processes as well as control probes. A total of 12 array hybridizations were performed, consisting of four biological replicates of the three different brain regions. After washing, slides were scanned for Cy3 and Cy5 fluorescence using Scanarray 5000 XL (GSI Lumonics, Kanata, Ontario, Canada), and quantified using GenePix Pro 4.0 software (Axon Instruments Inc., Union City, CA). Data were processed as described previously [34], and the results were expressed as the relative change (KO/WT). Genes were considered to be upregulated or downregulated if the ratio was at greater than 1.5 or less than 0.67, respectively. The microarray expression data could be studied from the NCBI-GEO, database entry GSE11596 (http://www.ncbi.nlm.nih.gov/geo/query/acc.cgi?accGSE11596).

### Quantitative real-time reverse transcriptase polymerase chain reaction (qRT-PCR)

Total RNA (1 µg) was reverse-transcribed using random primers with SuperScript™ II reverse transcriptase (Invitrogen Corp.). A negative control reaction was run to confirm the absence of genomic DNA contamination. Furthermore reverse transcription-PCR primers were designed between different exons to avoid any amplification of genomic DNA. In order to establish the most appropriate conditions for the reaction the quantity of sample template was tested. As a result, 25 ng of cDNA were used for each PCR amplification.

The quantitative real-time PCRs [79] were set up in a reaction volume of 20 µl. Each reaction mixture contained 9 µl of the experimental cDNA, 500 nM of each primer, and 10 µl of 2× SYBR Green PCR Master Mix (Applied Biosystems, Foster City, CA). Multiple negative water blanks were tested, and a calibration curve was determined in parallel with each analysis. All measurements were performed in triplicate and three biological replicates of the experiment were tested. Expression values were normalized with respect to *Gapdh* expression as an endogenous control. PCR reactions were run and analyzed using the 7900HT Sequence Detection System (Applied Biosystems) under the thermal cycling conditions recommended by the manufacturer. qRT-PCR was carried out in WT and KO samples from all brain tissues to quantify the following mouse genes: *Fkbp5*, *Mt1*, *Bai1*, *Mt2*, *Pcolce2*, *Irak1*, *Mobp*, *Prodh*, *Dlk1*, *Plagl1*, *Ddc*, *Gas5*, *Mllt2h*, *Txnip*, *Eya2*, *Cpm*, *Nudt9*, *Pxmp2*, *Ucp2*, *S100a9*, *Gtl2*, *Gprasp1*, *Mecp2e1*, and *Mecp2e2*. Primer sequences (Sigma-Aldrich, St Louis, MO) are listed in Supplementary Table 1.

To determine whether the expression of genes was significantly different between groups we applied Welch's t test [80], which takes into account unequal variances. In this particular case we compared WT and KO mice values for all the genes studied in each tissue separately.

### Chromatin immunoprecipitation

The parts of the brain from WT and KO mice were separately chopped into small pieces and incubated at 37°C for 1.5 hours in Hank's balanced salt solution (HBSS) with collagenase (300 U/ml). Homogenized tissues were washed twice in cold phosphate-buffered saline (PBS) containing protease inhibitors (cOmplete EDTA-free, Roche Diagnostics, Indianapolis, IN). For preparation of crosslinked chromatin, they were incubated in PBS with 1% formaldehyde for 15 min at RT. Crosslinking was stopped by adding glycine to 125 mM, and cells were washed in PBS. All subsequent procedures were performed on ice, with buffers containing the protease inhibitors (cOmplete EDTA-free) as previously described [21]. Sonication yielded chromatin fragments of 300–600 bp in length. Immunoprecipitations were performed using 5 µg of a rabbit polyclonal anti-MeCP2 [81]. The sensitivity of PCR amplification was tested using serial dilutions of total DNA collected after sonication (input fraction), and three independent experiments were performed for each analyzed promoter. The samples used for PCR amplification included total DNA (“Input”), a washing fraction of the no-antibody control (“NAB”), and the fraction immunoprecipitated by the antibody (Bound: “Mecp2 B”). We included an immunoprecipitation for total histone H3 to show that the chromatin purified from the KO animals was prepared exactly as that from wild-type animals. We also included a negative control IgG antiserum to show that the obtained signals were not due to a specific association of the Mecp2 antiserum to certain chromatin structures. After PCR, all products were run in a 2% agarose gel. Quantitative real-time PCR analysis was performed on an ABI 7900HT sequence detection system (Applied Biosystems, Foster City, CA) using SYBR® Green. The quantitative real-time PCR data were analyzed as described by Chahrour et al. (22). Primer sequences are listed in Supplementary Table 1.

### Bisulfite genomic sequencing

Genomic DNA was extracted from each mouse brain region, and bisulfite modification was carried out as previously described [82]. Primers for PCR amplification of the bisulfite-modified DNA were designed with Methyl Primer Express® Software (www.appliedbiosystems.com/methylprimerexpress), which ensures primer specificity for bisulfite-converted DNA and avoids CpGs in their sequence. PCR amplicons were purified with Ultra Clean GelSpin kit (MO BIO Laboratories, Inc.). PCR products were subcloned into the pGEM-T Easy Vector (Promega), and clones were picked in 96-well plates for sequencing (Montage Plasmid_96_ Miniprep Kit, Millipore). After a hot start (96°C for 1 min) and thermocycling (30 cycles of 96°C for 5 s, 50°C for 5 s, and 55°C for 4 min) using a BigDye Terminator V3.1 Cycle Sequencing Kit (Applied Biosystems), samples were purified using PERFORMA V3 96-Well Short Plate (EdgeBioSystems) apparatus, and then sequenced using a 3130×l Genetic Analyzer (Applied Biosystems).

CpG methylation status was determined for all gene promoters separately for the three different brain regions of WT and KO mice. Ten clones were analyzed from each of the six tissue combinations. Primer sequences are listed in Supplementary Table 1.

We carried out an ANOVA to examine possible effects of genetic condition of mice (WT vs. KO). Since the data are proportions, we used the standard [83] transformation arcsin(sqrt(p)), where p is the percentage methylation. We first modeled the proportion of methylation as a function of the genetic condition of mice, gene (since there could be differences in methylation related simply to being one gene or other), tissue (to account for any differences in methylation related to tissue), and two interactions – the gene×genetic condition (i.e., KO and WT might have different effects on methylation depending on the gene on which they were acting) and gene×tissue. In the eventm the type II sums of squares [84] indicated no evidence of significant interactions (gene×gene condition, p = 0.99; gene×tissue, p = 0.688), so these terms were dropped, to give a final model containing only the three main effects. Analyses were done using R [85] and the car library [86].

## Supporting Information

Figure S1 (0.11 MB PPT)

Figure S2 (0.12 MB PPT)

Figure S3 (0.67 MB PPT)

Figure S4 (0.10 MB PPT)

Figure S5 (1.51 MB PPT)

Table S1 (0.10 MB DOC)
