# Supplementary material for: Mecp2-Null Mice Provide New Neuronal Targets for Rett Syndrome
Source: PLoS One. 2008 Nov 7;3(11):e3669. doi: 10.1371/journal.pone.0003669 (PMC2576441; doi:10.1371/journal.pone.0003669)
Supplement: Figure S1 — (0.11 MB PPT) [file pone.0003669.s001.ppt]

## Slide 1
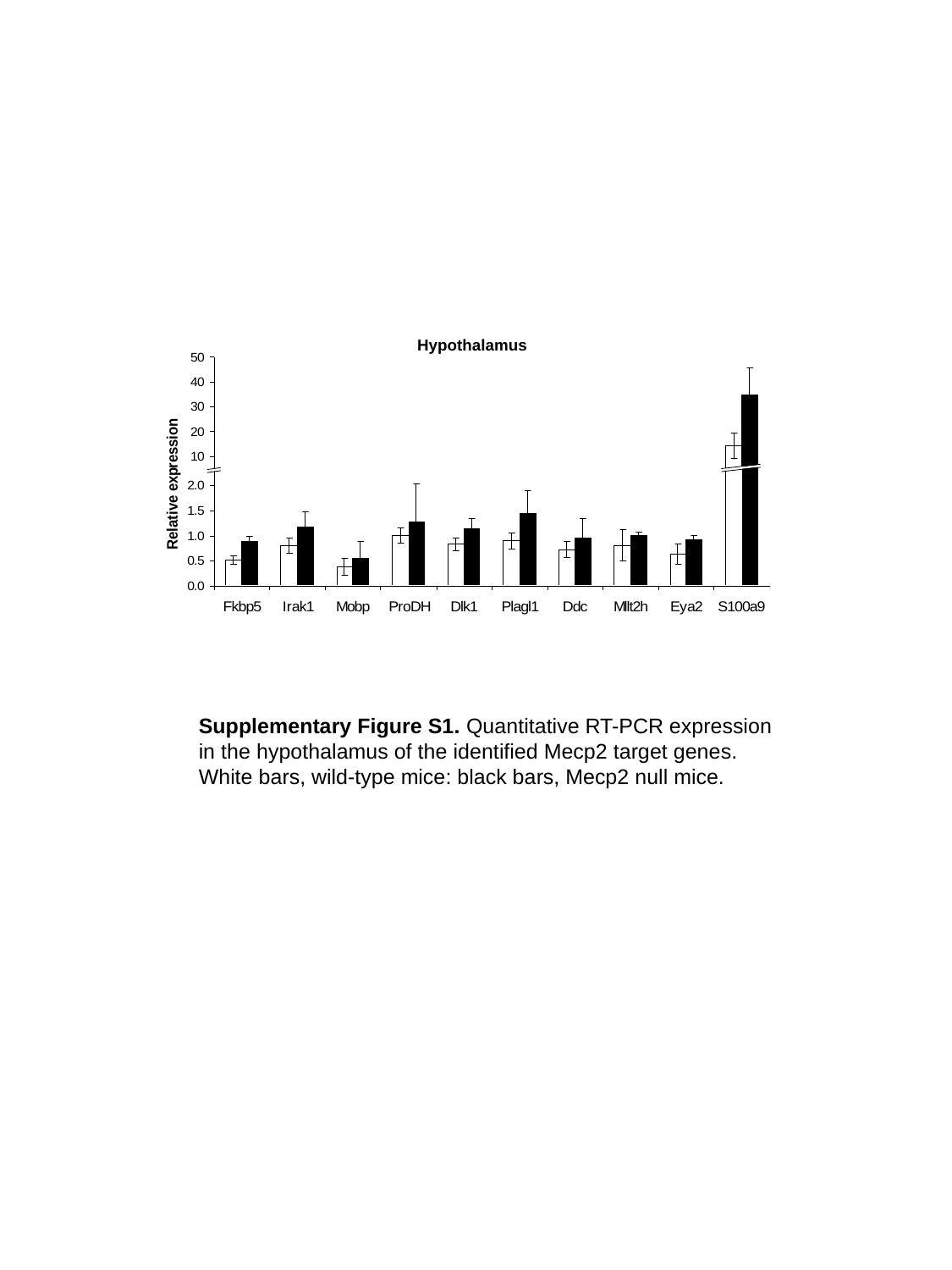

Hypothalamus
Supplementary Figure S1. Quantitative RT-PCR expression in the hypothalamus of the identified Mecp2 target genes. White bars, wild-type mice: black bars, Mecp2 null mice.
