# Supplementary material for: Mecp2-Null Mice Provide New Neuronal Targets for Rett Syndrome
Source: PLoS One. 2008 Nov 7;3(11):e3669. doi: 10.1371/journal.pone.0003669 (PMC2576441; doi:10.1371/journal.pone.0003669)
Supplement: Figure S2 — (0.12 MB PPT) [file pone.0003669.s002.ppt]

## Slide 1
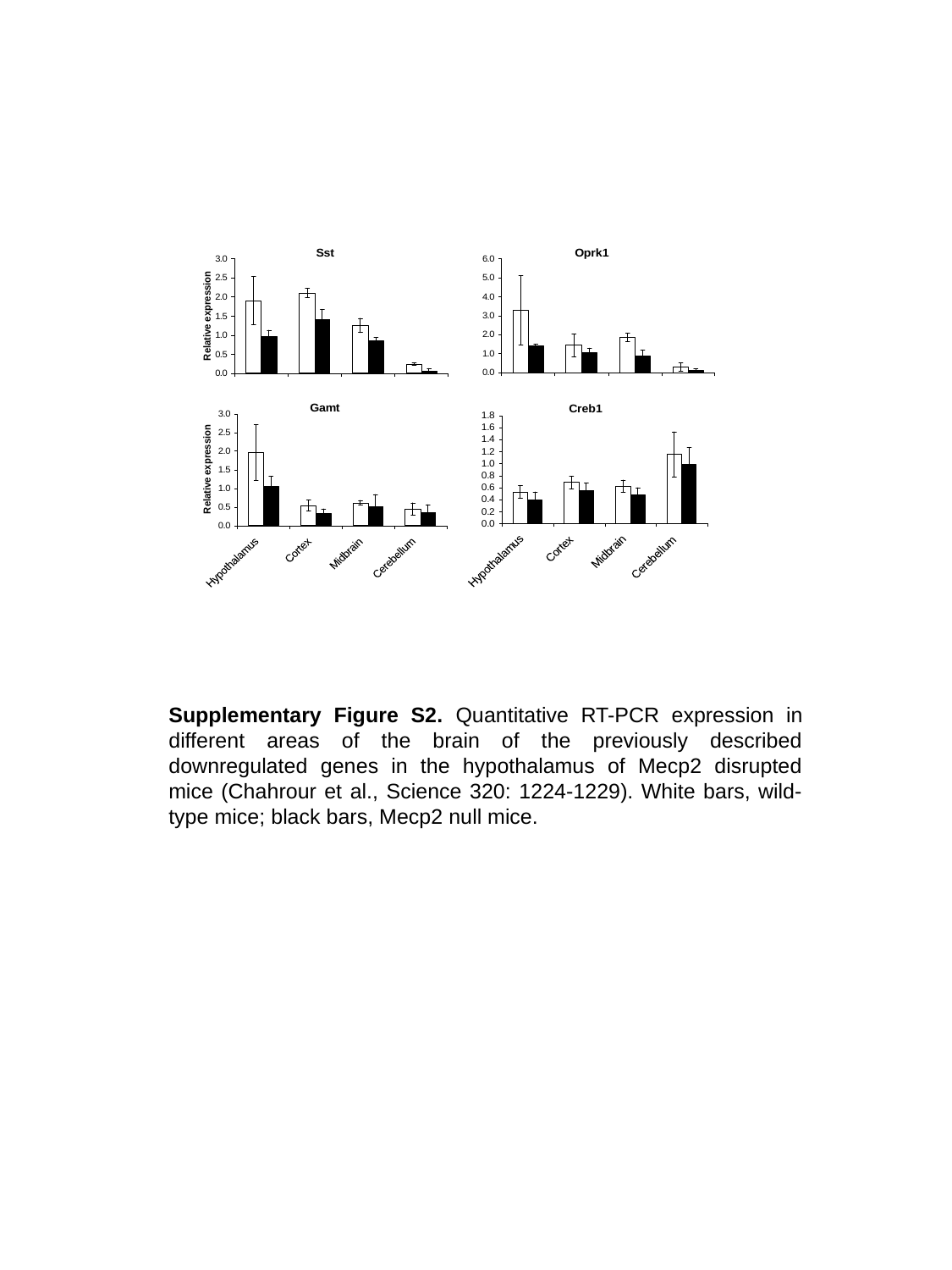

Supplementary Figure S2. Quantitative RT-PCR expression in different areas of the brain of the previously described downregulated genes in the hypothalamus of Mecp2 disrupted mice (Chahrour et al., Science 320: 1224-1229). White bars, wild-type mice; black bars, Mecp2 null mice.
