# Supplementary material for: Mecp2-Null Mice Provide New Neuronal Targets for Rett Syndrome
Source: PLoS One. 2008 Nov 7;3(11):e3669. doi: 10.1371/journal.pone.0003669 (PMC2576441; doi:10.1371/journal.pone.0003669)
Supplement: Figure S3 — (0.67 MB PPT) [file pone.0003669.s003.ppt]

## Slide 1
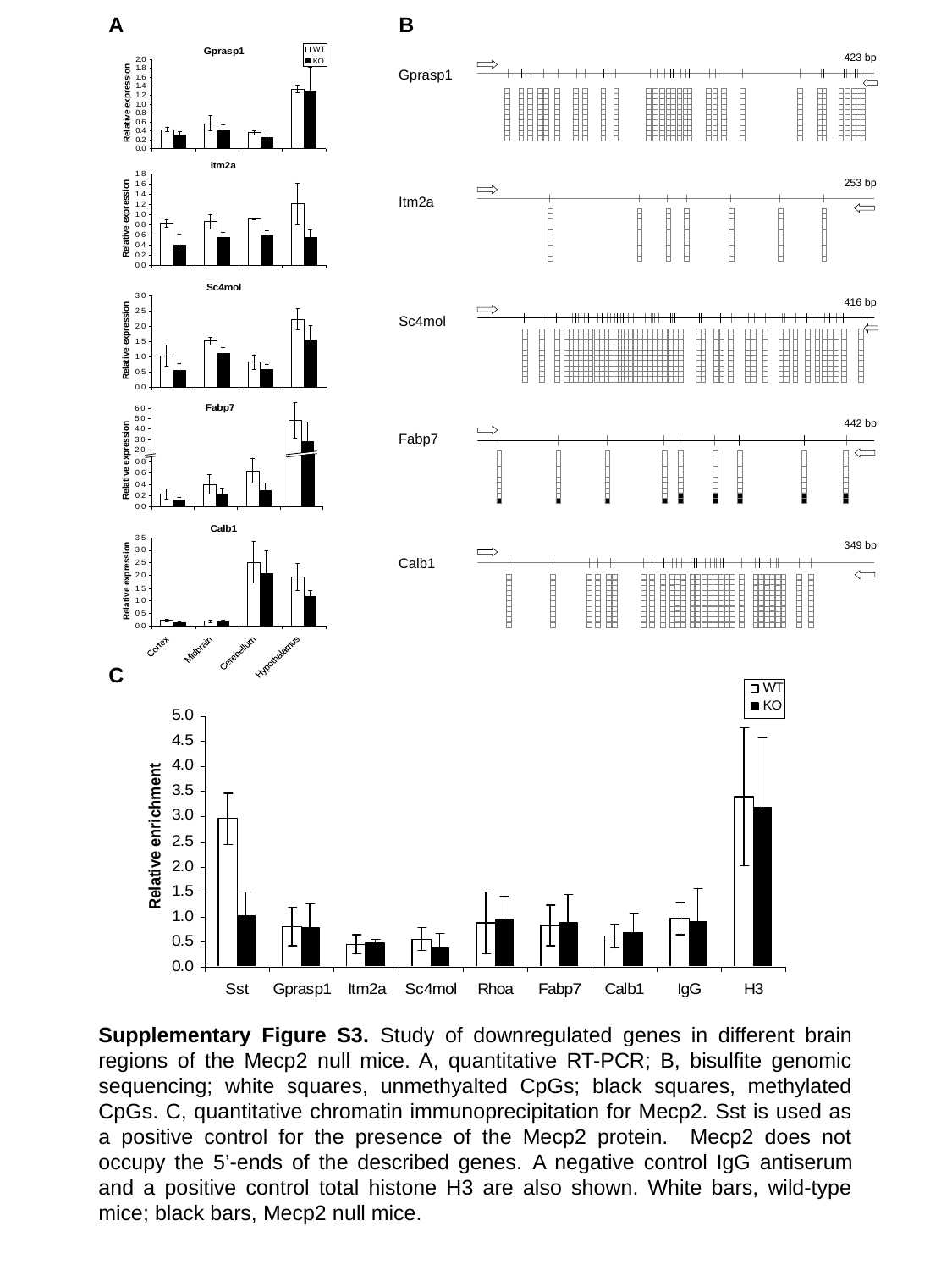

A
B
423 bp
Gprasp1
253 bp
Itm2a
416 bp
Sc4mol
442 bp
Fabp7
349 bp
Calb1
C
Supplementary Figure S3. Study of downregulated genes in different brain regions of the Mecp2 null mice. A, quantitative RT-PCR; B, bisulfite genomic sequencing; white squares, unmethyalted CpGs; black squares, methylated CpGs. C, quantitative chromatin immunoprecipitation for Mecp2. Sst is used as a positive control for the presence of the Mecp2 protein. Mecp2 does not occupy the 5’-ends of the described genes. A negative control IgG antiserum and a positive control total histone H3 are also shown. White bars, wild-type mice; black bars, Mecp2 null mice.
