# Supplementary material for: Mecp2-Null Mice Provide New Neuronal Targets for Rett Syndrome
Source: PLoS One. 2008 Nov 7;3(11):e3669. doi: 10.1371/journal.pone.0003669 (PMC2576441; doi:10.1371/journal.pone.0003669)
Supplement: Figure S4 — (0.10 MB PPT) [file pone.0003669.s004.ppt]

## Slide 1
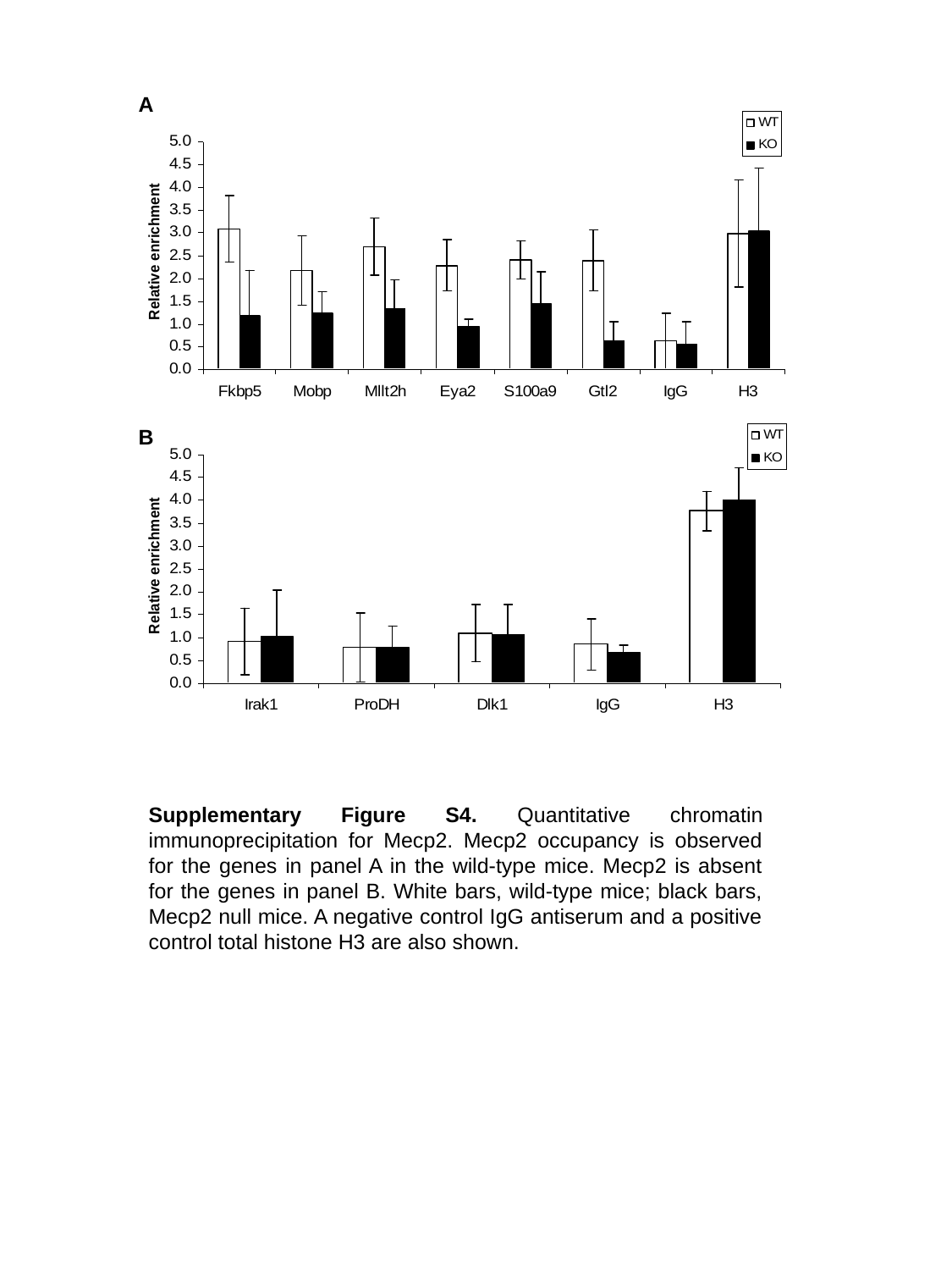

A
B
Supplementary Figure S4. Quantitative chromatin immunoprecipitation for Mecp2. Mecp2 occupancy is observed for the genes in panel A in the wild-type mice. Mecp2 is absent for the genes in panel B. White bars, wild-type mice; black bars, Mecp2 null mice. A negative control IgG antiserum and a positive control total histone H3 are also shown.
