# Supplementary material for: Mecp2-Null Mice Provide New Neuronal Targets for Rett Syndrome
Source: PLoS One. 2008 Nov 7;3(11):e3669. doi: 10.1371/journal.pone.0003669 (PMC2576441; doi:10.1371/journal.pone.0003669)
Supplement: Figure S5 — (1.51 MB PPT) [file pone.0003669.s005.ppt]

## Slide 1
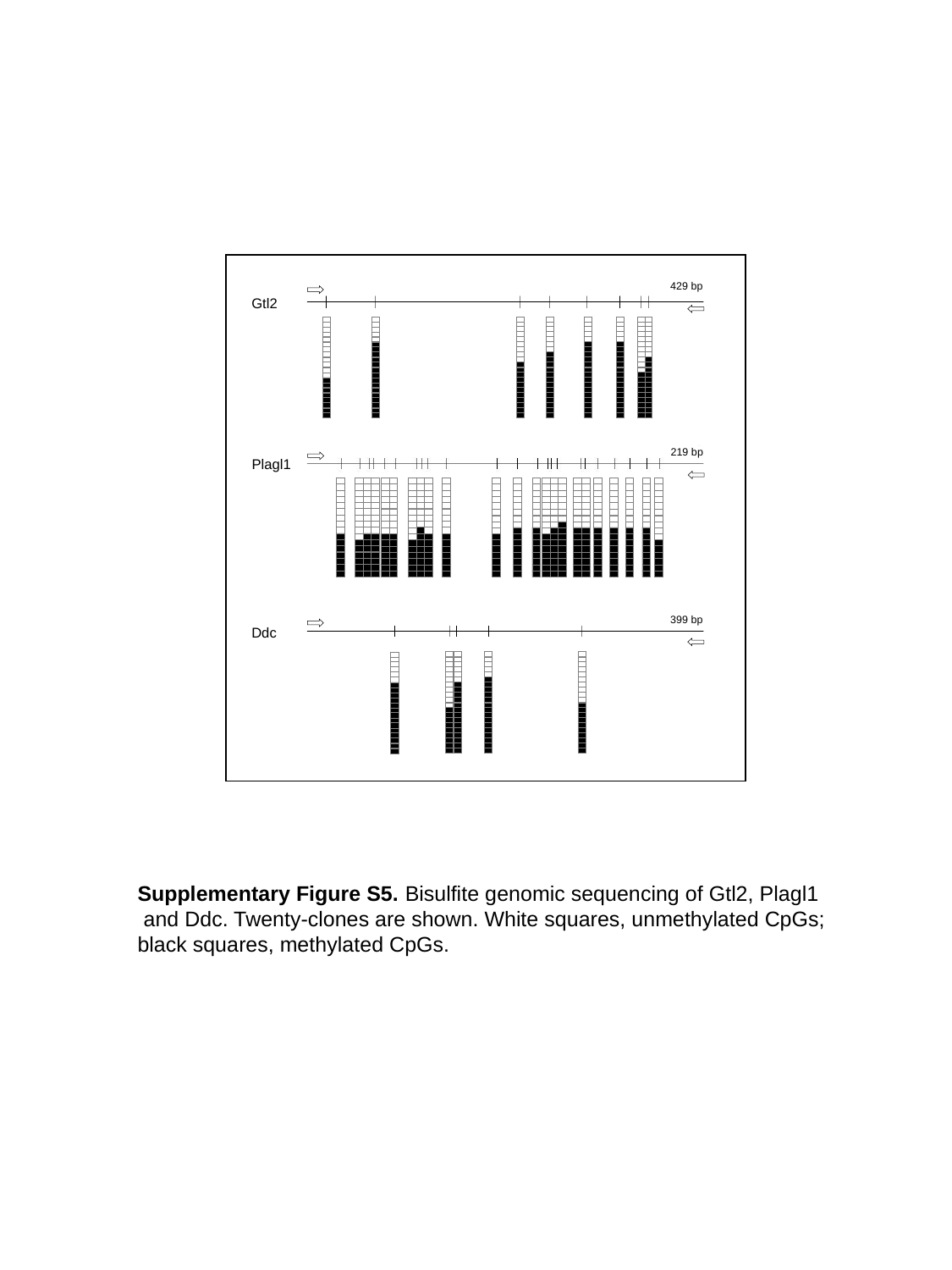

429 bp
Gtl2
219 bp
Plagl1
399 bp
Ddc
Supplementary Figure S5. Bisulfite genomic sequencing of Gtl2, Plagl1
 and Ddc. Twenty-clones are shown. White squares, unmethylated CpGs;
black squares, methylated CpGs.
