# Supplementary material for: Mecp2-Null Mice Provide New Neuronal Targets for Rett Syndrome
Source: PLoS One. 2008 Nov 7;3(11):e3669. doi: 10.1371/journal.pone.0003669 (PMC2576441; doi:10.1371/journal.pone.0003669)
Supplement: Table S1 — (0.10 MB DOC) [file pone.0003669.s006.doc]

**Suplementary Table 1.** Primers used for the assays.

| **Primers** | **Forward 5’ 🡪 3’** | **Reverse 5’ 🡪 3’** |
| --- | --- | --- |
| **RT** |  |  |
| Bai1 | CGACAGTAGCCCCAGCTC | GGGTTTCAGGAGACAGTGGA |
| Calb1 | GGAGCTGCAGAACTTGATCC | AAGCCGCTGTGGTCAGTATC |
| Creb1 | GTGTAGTTTGACGCGGTGTG | CTGGGCTAATGTGGCAATCT |
| Cpm | AAACCTGTGGGTTCTCGTTG | TAACAGTCGGGCTTCTGGAC |
| Ddc | CTGATGTGGAGCCTGGCTAT | GAGAAACCAATGCAGCCAAT |
| Dlk1 | TGTGACCCCCAGTATGGATT | CTTTCCAGAGAACCCAGGTG |
| Eya2 | GTGTGCCTCAGCTCTTTGTG | TAGGTGTAGGGGCTCTGTCC |
| Fabp7 | GAAGGTGGCAAAGTGGTGAT | ACAGCAACGATATCCCCAAA |
| Fkbp5 | GGGCACCAGTAACAATGGAG | GGCAAATGGCTTCTTTCTGT |
| Gamt | GAGTGCAATGATGGGGTCTT | CGTGAGGTTGCAGTAGGTGA |
| Gapdh | GGAGCCAAACGGGTCATCATCTC | AGAAGACTGTGGATGGCCCCTC |
| Gas5 | GAGTGGGTGGGAAGTCTGAA | AATGAACAAGCATGCAACCA |
| Gprasp1 | CACCACTTGAACGCAGAGAA | AGGATAGCTCTGCACGACGA |
| Gtl2 | TTGCTGTTGTGCTCAGGTTC | GTGTCTGTGTCCGTGTGTCC |
| Irak1 | GACCAAGTATTTGAAAGACCTG | GTAGTGCCTCCCTGGGTACA |
| Itm2a | TGGAAGACTGGGAGAGGAGA | TCCCAGATGAGCCATCTTTC |
| Mecp2_e1^*^ | AGGAGAGACTGGAGGAAAAGT | CTTAAACTTCAGTGGCTTGTCT |
| Mecp2_e2^*^ | CTCCATAAAAATACAGACTCACCAGT | CTTAAACTTCAGTGGCTTGTCT |
| Mllt2h | GGAGTCAGAGAGCAGCGAGT | GGGAGAGGGTCTTTGGAATC |
| Mobp | AACTCCAAGCGTGAGATCGT | CAGAGGCTGTCCATTCACAA |
| Mt1 | CTCCGTAGCTCCAGCTTCAC | GTTCGTCACATCAGGCACAG |
| Mt2 | CCGATCTCTCGTCGATCTTC | ACTTGTCGGAAGCCTCTTTG |
| Nudt9 | GCCAAAGAGAATTCCCACAA | TGGGTCTTCCGTTTTCAATC |
| Oprk1 | TACCTGCTCTCCCAGTGCTT | GTTGGTTGCGGTCTTCATCT |
| Pcolce2 | TCGCTATGACTTCGTGGATG | CTGGCCAGTTGGGAGTTTTA |
| Plagl1 | ACCCAGCTATTGTGCTCTGG | ACACTCAGCCTTCGAGCACT |
| Prkca | AGAAGGGCACATCAAAATCG | TTGGACAAGGATTTGGGGTA |
| Prodh | CCGTGGACTTCACCAACAC | TCCAGGATAAAGCCAACACC |
| Pxmp2 | AGACGATTGAGAAGCGGAAA | AAGGTGTCCACATCCTCCAG |
| S100a9 | TCATCGACACCTTCCATCAA | TTACTTCCCACAGCCTTTGC |
| Sc4mol | GGCTCCCTGATAGTTCACGA | TGGCATTCTTTCCCAATCAT |
| Sst | CCCAGACTCCGTCAGTTTCT | GAAGTTCTTGCAGCCAGCTT |
| Ttr | CAGATCCACAAGCTCCTGAC | CAGGATCCCTCAGAGGTCTTT |
| Txnip | TTTGAGGTGGTCTTCAACGA | AGCTCGAAGCCGAACTTGTA |
| Ucp2 | CGTCTCCCAGCCATTTTCTA | ACATCTGTGGCCTTGAAACC |
| **ChIP** |  |  |
| Bai1 | GTGGGGAGGAGCCTGAGAG | CTTCGGAGCCGTTCTTACCT |
| Calb1 | CCACCTGCTGCTTCCTAGAC | CCGCACCCAGTTCTCTGTAT |
| Ddc | TGGGATTCCAGGAAAAGACA | CATTCTGGCCCACATTCTG |
| Dlk1 | TCTTCCACATCTTCGTGGTG | CGCACTCTTTGGCTAGACG |
| Eya2 | CGGGACTCTCTCTCATTCCA | CACCACAGCGATTCAAACAC |
| Fkbp5^**^ | GCACAATGCTGGACTAGATA | TTTAATGTGCTCAGTGCTTC |
| Fabp7 | GGGTAAGACCCGAGTTCCTC | AAATCGGGGGTTTCTAAGGA |
| Gprasp1 | ATTTGGTAATCGCCTGCTTG | TCACCTTTCGGTACCCACTC |
| Gtl2 | TTGGGGTAGTGGGGACATTA | AGAAAGTGCCCTGGAAATGA |
| Irak1 | GGGCCATTTTGTTACCCATA | GGGAGTCCCTAATGGGAGAA |
| Itm2a | CCTGGGACTGCGTCATAAGT | ACTGACGAGCGCCTCTACAT |
| Mllt2h | TTTTTCTTTTGTGGCCTTGG | CTCACTCGTGACCCCTGACT |
| Mobp | GGGCCTTTGTGTAGCTTTTG | TGATAGCTTCCCCTGTGACC |
| Mt1 | AGCAGCGATAGGCCGTAATA | GGTGACGCTTAGAGGACAGC |
| Mt2 | CAGAGAAAGGGGGTGTGACT | ATCGACGAGAGATCGGTTTG |
| Plagl1 | TCTGCGATTTGTCACTCAGC | GCCCCGGTTGTCACTACTTA |
| Prodh | GGAAGTACGCCAAACCAGAG | CTTGCGGTTTGGTTGAGAG |
| S100a9 | CCATTGGGAAAGAGCTACCA | CTCACTTCCCTTCCATCCAA |
| Sc4mol | GCTAGGGCTTCGTTGACTTG | GGCTAGAGAGCGCCTTACCT |
| Sst^***^ | GCGTAAAAGCACTGGTGAGATCT | GGTCTCCCCTTTTTAAACTCTCTCTCT |
| **BS** |  |  |
| Bai1 | TTTTTAGGAAGGGAAAAAAGG | TTAATCCAACRACTTACAAAACC |
| Calb1 | TTAGTTATTTTTTGGGAAGAGA | ATAAAACCAAATCTCAAAAAACT |
| Ddc | AGGTAAAGGTGTAGGTTATTAGGAG | CCACACAATACTACACAATAACCAC |
| Dlk1 | AAGTGTTTTATTGTGTTTGTGAG | AAACCAAAATTACACCRAA |
| Eya2 | AGGGTTTGGTTTAGATTTGAA | AACAACCAAACCTCTACAAAAA |
| Fabp7 | ATTGGGAGGATTTTGATTTATT | CTCAACCTATCTTTCAAAAACCT |
| Fkbp5 | GTTTTTGTTTTGTTGTGTTGTT | AAACAAACTATTCTCAAACAAACA |
| Gprasp1 | GGAAAGATATGTTTAAGAAGGAAGT | CACACAAAAAAAAAACAAACAA |
| Gtl2 | GGTGTTTTTTTTTGGAATGAGT | CCCCCTTTAACACATAAATAACA |
| Irak1 | GTTTGAAGGGGAAGTGAGTTAG | AAAACATCAAACTTCAACTCCA |
| Itm2a | GTAGGAGTATGTTTGGGGTGTA | AATATCTCCTCTCCCAATCTTC |
| Mllt2h | TGGAGTTAGATTTGTAGGTGTG | ACCTCCTTCTCCTAATAAACTTCT |
| Mobp | TTTGTTGGTTTTTGTTTTTTTT | ACTTCCCCTATAACCCATAAAA |
| Mt1 | GGGAAAGTATTATAGGGATATGATG | AAAAACAACCTACCCTCTTTATAATC |
| Mt2 | TTTGAGTTTAGAGAAAGGGGGTGTG | ACCAAAAAACCTATCTAACTCTCCC |
| Plagl1 | GTYGTTATGGTTGTTTAGGTTG | CACCCAAATTCAAAATTTATCA |
| Prodh | GGYGTGGTTAATTTAGTGTATTT | CCCAACTTCAAACCATACTTAA |
| S100a9 | GGGAAAGAGTTATTATTTTTGAGG | ATTAACTCACTTCCCTTCCATC |
| Sc4mol | TTGGATGAAGTTAAGTTAATGTGA | AATACAACRACCTCCTCACTAC |

^*^ From the articles:

- Dragich JM, Kim YH, Arnold AP, Schanen NC (2007) Differential distribution of the MeCP2 splice variants in the postnatal mouse brain. J Comp Neurol 501: 526-542.

- Mnatzakanian GN, Lohi H, Munteanu I, Alfred SE, Yamada T, et al. (2004) A previously unidentified MECP2 open reading frame defines a new protein isoform relevant to Rett syndrome. Nat Genet 36: 339-341.

^**^ From the article:

Nuber UA, Kriaucionis S, Roloff TC, Guy J, Selfridge J, et al. (2005) Up-regulation of glucocorticoid-regulated genes in a mouse model of Rett syndrome. Hum Mol Genet 14: 2247-2256.

^***^ From the article:

Chahrour M, Jung SY, Shaw C, Zhou X, Wong ST, Qin J, Zoghbi HY (2008) MeCP2, a key contributor to neurological disease, activates and represses transcription. Science 320:1224-1229.

(RT= qRT-PCR; ChIP = Chromatin immunoprecipitation; BS = Bisulfite sequencing).
